# Plasmonic Optical Biosensors for Detecting C-Reactive Protein: A Review

**DOI:** 10.3390/mi11100895

**Published:** 2020-09-27

**Authors:** Joo Seon Seok, Heongkyu Ju

**Affiliations:** 1 Department of Physics, Gachon University, Seongnam-si, Gyeonggi-do 13120, Korea; wntjs0807@gmail.com; 2 Gachon Bionano Research Institute, Gachon University, Seongnam-si, Gyeonggi-do 13120, Korea

**Keywords:** C-reactive protein (CRP), optical biosensor, surface plasmon, nanoparticle

## Abstract

C-reactive protein (CRP), a potent acute-phase reactant that increases rapidly in response to inflammation, tissue damage or infections, is also considered an indicator of the risk of cardiovascular diseases and neurological disorders. Recent advances in nanofabrication and nanophotonic technologies have prompted the optical plasmonic phenomena to be tailored for specific detection of human serum CRP into label-free devices. We review the CRP-specific detection platforms with high sensitivity, which feature the thin metal films for surface plasmon resonance, nano-enhancers of zero dimensional nanostructures, and metal nanoparticles for localized surface plasmon resonance. The protocols used for various types of assay reported in literature are also outlines with surface chemical pretreatment required for specific detection of CRPs on a plasmonic surface. Properties including sensitivity and detection range are described for each sensor device reviewed, while challenges faced by plasmonic CRP sensors are discussed in the conclusion, with future directions towards which research efforts need to be made.

## 1. Introduction

In 1930, Tillett and Francis discovered the protein that responded to the encapsulated (c)-polysaccharide of pneumococcus in patients with acute pneumococcal infections [1], later named the C-reactive protein (CRP). In blood sera of healthy individuals, CRP is known to be present usually in a small amount. CRP production in the circulation elevates its level in serum in response to various inflammations, infection, stress, trauma, surgery, tissue damage, cardiovascular disease, and neurological degeneration [2,3,4,5,6,7,8,9,10,11,12,13]. During an acute phase response, the CRP level in serum increases rapidly (by more than 100-fold) and reaches a peak within 48 h with its half-life of about 19 h [9,14,15,16,17,18,19,20,21].

The CRP levels in blood plasma are considered to be lower than about 5 μg/mL for individuals with no inflammation. However, the small variation of the level below it may depend on onset of other ailments such as cardiovascular diseases (CVD). It is seen that the advent of a high-sensitivity immunoassay of CRP allows the use of the risk predictive characteristic of its levels for evaluating CVD such as acute coronary syndromes [2,14,15,16,17,22,23,24,25]. The American Heart Association (AHA) and the US Centers for Disease Control and Prevention (CDC) defined the CVD risk groups using CRP levels as follows [26,27]: low risk, CRP concentration <0.1 μg/mL; moderate risk, CRP concentration 1.0–3.0 μg/mL; and high risk, CRP concentration >3.0 μg/mL.

CRP mainly produced from hepatocytes in the liver, can also be produced in smooth muscle cells, macrophages, endothelial cells, lymphocytes, and adipocytes. CRP has a pentameric structure of the five identical monomers combined, moves through the blood to the region where inflammatory reaction occurs, and then irreversibly dissociates into the monomers that play a direct role in the inflammatory response [14,15,16,17,28]. The mechanisms behind the CRP variants’ generation needs to be clarified to deal with various abnormalities such as acute coronary syndromes. This also addresses the requirement of developing ultra-high sensitivity biosensors for CRP detection [29].

Highly sensitive CRP detection performed mostly in vitro exploits the immunoreaction-based methodologies such as the turbidoimmunometric assays [30,31,32], immunonephelometric assays [33,34,35,36], the enzyme-linked immunosorbent assay (ELISA) [37,38], the fluorescence assay with immunoreaction [39,40,41], and the optical plasmonic immunoassays [19,29,42,43,44,45,46,47,48,49,50,51].

Amid the methodologies mentioned above, optical plasmonic sensors have attracted much attention for detecting biomarker molecules without labels such as fluorescent tags, due to a number of advantages [52,53,54,55,56,57,58,59]. They include the high sensitivity without contaminating target biomolecules, the capability of multiplexed assay, a small amount of an analyte sample required, the relative immunity to external factors (ambient temperature variation/external electric disturbance), and the observability of time-dependent kinetic behavior of bio-molecular interactions [3,60].

In particular, the high sensitivity in optical plasmonic sensors arises from locally enhanced density of photonic modes near where plasmons are excited [56,61,62,63,64,65,66]. When bringing target biomolecules of non-negligible molecular weight such as CRP molecules (molecular weight of 118–144 kDa [67]) into close proximity with a plasmonic metal surface, their strong interaction with the plasmonic local fields modifies the optical resonance condition via the effective refractive index change. This thus produces the drastically altered value of the optical output coupled into the medium that allows photon momentum conservation [68,69,70,71,72]. This kind of a detection platform enables only the target molecules near the metal surface to be detected, forming the basis of plasmonic biosensors of any kind which can excite either localized surface plasmon resonance (LSPR) or propagating surface plasmon resonance (SPR).

This paper reviews the CRP-detecting immunoassays relying on optical plasmonic resonances. Section 2 presents the SPR-based CRP immunoassays, while Section 3 the LSPR-aided CRP immunoassays. Various protocols on the basis of immune reaction for specific detection of CRP such as direct assays, sandwich assays and sandwich assays with nano-enhancers, are described with the relevant surface chemistry accessible to the plasmonic surface. Characteristics of each CRP assay are also outlined for comparison, including the detection limit and range associated with the given plasmonic transducers.

## 2. Surface Plasmon Resonance-Based C-Reactive Protein (CRP) Detection

SPR biosensors detect changes in an effective refractive index of dielectric media on the sensing surface, which is caused by adsorbing biomolecules on it. The analyte biomolecules need to be adsorbed within the penetration range of plasmonic evanescent fields from the surface, the interface between the nanometer thickness metallic film and the dielectric media [61,73]. SPR can occur at the interface between metal and dielectric media that include target biomolecules (e.g., CRPs), via a coherent coupling of surface plasmons with external photons. The surface parallel component of photon momenta offered by a high index prism [19,29,42,43,44,45,46,47,72,74] or a fiber core [48,55,75,76,77,78,79], can match those of surface plasmons, generating a coherently coupled state, i.e., the surface plasmon polaritons. The momenta required for such matching are subject, with great sensitivity, to a change in an effective refractive index of dielectric media near a metal film, due to the Debye screening effects, thus overriding the resonance conditions [61,80,81]. This serves as the basis for detecting, with high sensitivity, biomolecules adsorbed on the metal surface. SPR biosensors typically interrogate a wavelength or an angle of incident light to pick up the reflectance dip, i.e., the so-called “plasmonic absorption”. The absorption peak shifts in a wavelength or in an angle, as a function of effective refractive index, its sensitivity being dominated by a complex dielectric constant of the metal film used. Meanwhile, the peak broadening caused by metallic ohmic loss and Landau damping of surface plasmons determines the sensor resolution [54,61,73,82,83,84].

**Figure 1:**
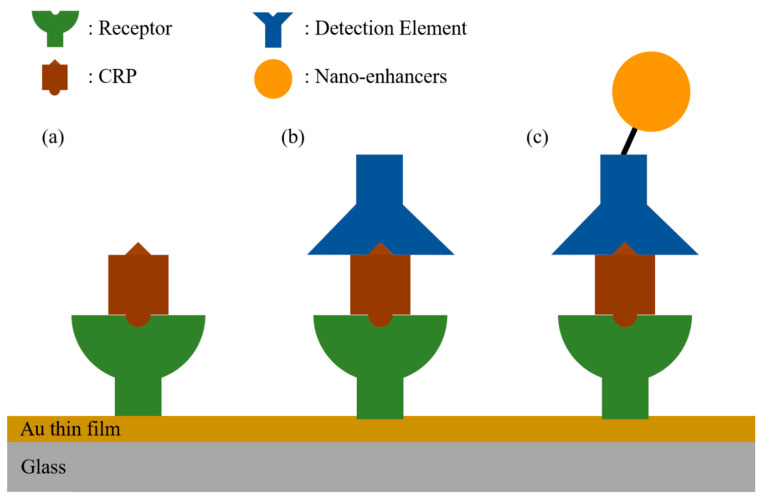
Schematic of three strategies for the surface plasmon resonance (SPR)-based C-reactive protein (CRP) sensors. (**a**): a direct immunoassay, (**b**): a sandwich immunoassay, (**c**): a sandwich immunoassay with nano-enhancers such as nanoparticles, and quantum dots (QDs).

Figure 1 illustrates the three protocols used to build the SPR immunoassay chip structures for CRP detection [19,29,42,45]. Gold (Au), the chemically stable metal, is widely used for a plasmonic film due to the great availability for biochemical surface treatment required prior to target-specific reaction. The three protocols all need bio-recognition receptors to capture CRPs selectively, even amid the presence of many other kinds of biomolecule. The receptor immobilization requires the prerequisite surface treatment such as via surface functionalization into a self-assembled monolayer (SAM) [42,43,48], through biotin-streptavidin/extravidin interaction [19,44,46] and via the G proteins [29], via the protein adhesive films [45], and using thiol-modified receptors [47]. Upon the receptor immobilization, injection of CRPs onto the sensing surface leads to selective capture as seen in Figure 1a, and in turn induces change in a SPR signal, being referred to as a direct immunoassay in SPR sensors [29,42,44,45,48]. Subsequent injection of detection elements such as the detection antibodies (dAbs) or aptamers, as seen in Figure 1b, amplifies the SPR signal together with escalating selectivity in detecting CRP, as referred to as a sandwich immunoassay in SPR sensors [19]. Further enhancement of sensitivity in CRP detection can be realized via replacing the detection elements by nano-enhancer-conjugated detection elements such as Au nanoparticles conjugated antibodies [47] or quantum dots (QDs) conjugated aptamers, as seen in Figure 1c [46]. It is noted that, all substances injected consecutively in a sandwich assay, which are supposed to induce the effective index change near metal surface, need to fall within the penetration range of plasmonic evanescent fields (~200 at visible wavelengths).

We organize Section 2 into three sections according to the immunoassay types used in the SPR-based plasmonic sensing platforms, as follows: Section 2.1 covers the direct assay, Section 2.2 the sandwich assays and Section 2.3 the nano-enhancers in a sandwich assay.

### 2.1. Direct Immunoassay

Casa et al. [42] used the chemical bonding via 4,4′-dithiodibutyric acid (DDA) to immobilize CRP antibodies on the Au surface. Through its disulfide bond, DDA attached to the surface, with a consequence of the carboxyl group modified Au surface. The amino group of the CRP antibody, in turn, bonded with it through 1-ethyl-3-(3-dimethylaminopropyl)carbodiimide hydrochloride-*N*-hydroxysuccinimide (EDC-NHS) crosslinking. The biosensor chip was tested with various concentrations of CRP ($10^{-1}-10^{2}$ µg/mL) to obtain the SPR angle shifts. The angle shift began at the CRP concentration of 1 µg/mL and plateaued at concentrations higher than 10 µg/mL. The SPR biosensor had a detection range of 1–10 µg/mL and a limit of detection (LOD) of 1 µg/mL. Comparison was also made between outcomes from using different flow cell shapes in the SPR chip. The flow cell type A had a circular shape, whereas type B was rectangular, and both types had the same depth of 10 µm. It was found that the rectangular flow cell (type B) resulted in a higher signal shift. An SPR analyzer (NTT Advanced Technology Corp., Kanagawa, Japan) was used for their SPR system. The sensor chips were made by sputtering Au on the glass plate and polydimethylsioxane (PDMS) was used to make the flow cell by a photolithography method.

**Figure 2:**
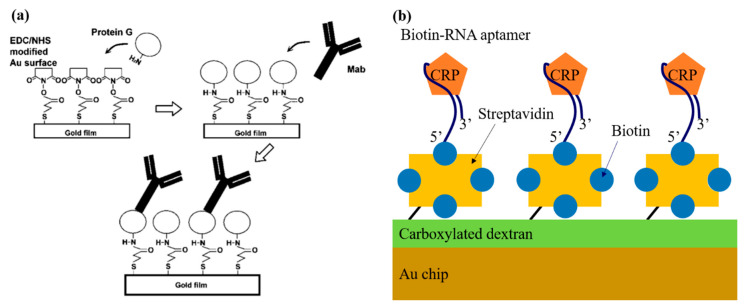
(**a**) Schematic of immobilizing antibody on protein G layer. Reprinted from [29], Copyright 2006, with permission from Elsevier. (**b**) Schematic of the sensing surface of the RNA-aptasensor for specific detection of CRP.

The CRP biosensor developed by Hu et al. [29] also relied on the direct immunoassay with an SPR-based signal transducer. Figure 2a illustrates the schematic of the antibody immobilizing model using the G proteins. The G protein has a Fc-binding domain that can interact with the Fc portion of IgG and thereby hold onto the antibody. After the Au surface of the chip had been functionalized with 16-mercaptohexadecanoic acid, the carboxyl group of the Au surface and the amine group of the G protein were conjugated through EDC–NHS crosslinking. Then, the CRP IgG antibody was immobilized by bonding with G protein. In this study, three different monoclonal mouse antibodies were used to differentiate between pCRP and mCRP; namely, antibody C8 to detect both pCRP and mCRP, antibody 8D8 to detect only pCRP, and antibody 9C9 to detect only mCRP. Each process was monitored in real time by measuring the SPR angle shifts. The SPR angle shifts were also observed when introducing pCRP of 0.226 µM (26 µg/mL) and mCRP of 0.437 µM (10 µg/mL) upon the three different antibodies used. With the antibody C8, the sensor could detect both types of CRPs. However, while the SPR angle shift by pCRP remained unchanged after surface washing, while those by mCRP decreased noticeably. Meanwhile, for 8D8 or 9C9 antibodies, only one of both types of CRPs produced a significantly detectable signal, although its sensitivity substantially lower than that for C8. This encouraged the use of the C8–pCRP immunoreaction pair for detecting the CRP level ranging from 1 µg/mL to 26 µg/mL with a LOD of 1 µg/mL [29]. This detection range appeared to meet the AHA and CDC criteria for clinical diagnosis of CRP.

Bini et al. used aptamers as the bio-recognition receptors that were believed to be more cost-effective and more stable than antibodies for a SPR-based CRP biosensor [44]. The schematic of their system is illustrated in Figure 2b. The biotinylated RNA aptamers were immobilized on the Au surface of the sensor chip, which was pretreated with layers of dextran, and streptavidin. The RNA aptamer sequence was selected through 10 Systematic Evolution of Ligands by Exponential enrichment (SELEX) rounds. Two types of aptamers, both biotinylated at the 5ʹ end but having different spacers (i.e., one with a 20mer polyT tail and the other with a triethylene glycol (TEG) tail), were tested. CRP binding curves for both cases exhibited the LOD of 5 ng/mL and the detection range of 5 ng/mL to 100 ng/mL. The reusability of both aptamers using HCl and NaOH were also demonstrated without losing sensitivity.

Choi et al. [45] immobilized antibodies using an oxygen plasma-treated parylene N film, and enhanced the sensitivity of the SPR-based sensor. The parylene N films treated with various oxygen plasma conditions were compared with a polystyrene substrate in terms of their ability to attach proteins such as bovine serum albumin (BSA). Various oxygen plasma conditions such as power levels and processing time for the parylene N film were tested. This enabled the optimized condition to be found for the most amplified SPR signal, i.e., 100 W for 1 min. Following CRP antibody immobilization onto the film, CRP concentrations of 1, 10, 100 ng/mL and 1 µg/mL were tested with this biosensor. The CRP concentrations and SPR signals appeared to be in a linear relationship. The biosensor with the plasma-treated parylene N film had a significantly better sensitivity than those with the bare gold surface of the chip or with the non-plasma treated film.

Optical fibers were introduced as the detection platform for SPR-based CRP assays by Aray et al. [48]. The fiber used as a sensor head comprised a 980 µm diameter core made of poly(methyl methacrylate) and 20 µm fluorinated polymer cladding. The gold film for creation of the SPR was formed on a flat surface into which the fiber core was polished along a 10 mm length. Unlike a typical prism based SPR sensor, the fiber SPR sensor does not need any precision device that can tune an incident angle of light for SPR excitation [85,86], but may be suitable for application where a miniaturized detection platform is required without compensating for the sensitivity at low cost. In a way similar to what was described above, the gold surface was first carboxylated using 11-mercaptoundecanoic acid, and the antibody was then immobilized to the carboxylated gold via the EDC–NHS reaction. The SPR signal was obtained by measuring the absorption peak shift of the spectrum of light transmitted through the fiber. CRP concentrations ranging from 0.01 µg/mL to 500 µg/mL were tested. The red shift of the absorption peak wavelength occurred with increasing the concentration, producing the LOD of 9 ng/mL.

### 2.2. Sandwich Immunoassay

**Figure 3:**
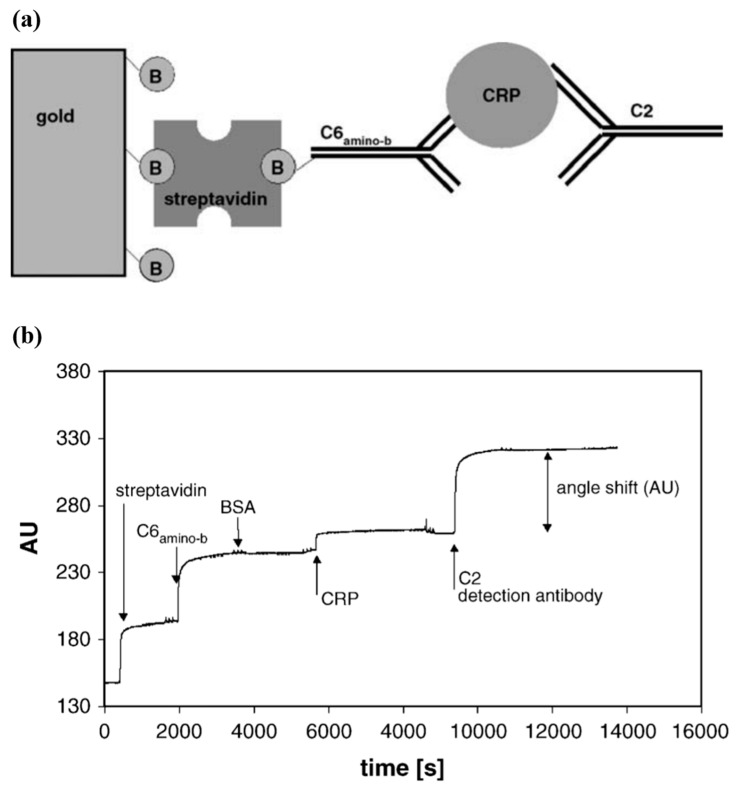
(**a**) Schematic of SPR chip (B, biotin; C6_amino-b_, biotinylated antiCRP C6; C2, antiCRP C2) (**b**) Detection principle-SPR sensorgram of the CRP sandwich assay. Reprinted from [19], Copyright 2006, with permission from Elsevier.

The first SPR-based immunosensor for CRP detection was reported in 2006 [19]. The SPR sensor system relied on a typical SPR spectroscope. As shown in Figure 3a, the gold surface of the sensor chip was treated with (3-aminopropyl)triethoxysilane to generate amino groups, and the biotin–NHS was subsequently used to create a biotin layer. The biotin layer then reacted with the streptavidin added prior to injection of biotin-labeled CRP capture antibody (cAb). The biotin-labeled cAb reacted with streptavidin, being fixed onto a chip surface. Injection of CRP caused the signal change due to its immunoreaction with cAb. Another signal change occurred due to the detection antibody (dAb) that was additionally injected to bind with CRP via another immunoreaction, ultimately forming a sandwich bonding. Figure 3b shows the continuously monitored signal changes during all procedures of biochemical molecules injection mentioned above. The SPR signal change from the dAb (C2) was greater than that caused by the CRP. This indicates that use of dAb allowed the enhanced sensitivity together with enhancing selectivity. Of the CRP concentrations (1–5 µg/mL) tested, the range of 2–5 µg/mL showed the nearly linear relationship of CRP concentration with the SPR signal with LOD of 1 µg/mL.

### 2.3. Nano-Enhancers for Sensitivity Enhancement

**Figure 4:**
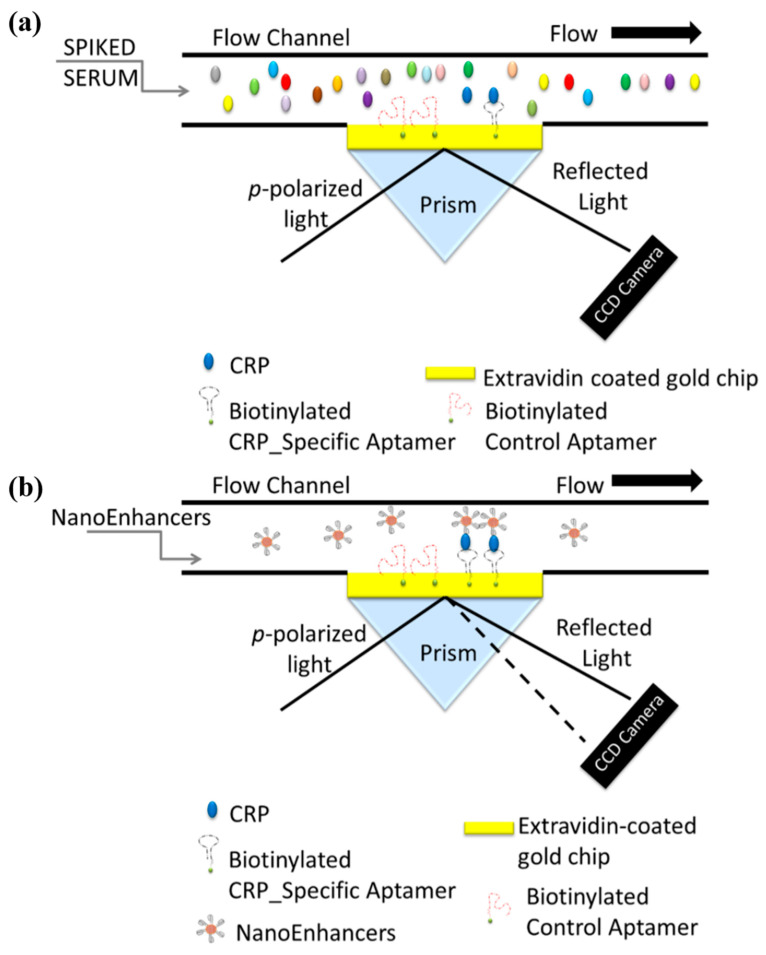
A schematic illustration of the sandwich protocol implemented for the detection of CRP in biological fluid. The gold-coated prism is prefunctionalized with aptamers specific to CRP and control aptamers followed by the (**a**) direct detection of CRP (fg/mL) spiked in human serum and the (**b**) sandwich based assay using CRP-specific aptamer-coated QDs for SPRi signal amplification. Direct detection of CRP (fg/mL) does not generate a quantifiable sensor response as depicted with no change in the angle of reflectivity, however, with sandwich assay the nano-enhancers induce a change in the reflectivity. Reproduced with permission from [46].

Vance and Sandros [46] designed the aptamer-coated QDs for use as detection elements in a sandwich assay to detect CRP. QDs conjugated with CRP-specific aptamers enhanced the SPR signal change significantly, thus QDs are referred to as nano-enhancers in this assay. The schematic of the sensing system is illustrated in Figure 4. The immobilization of the aptamers that were biotinylated required the gold surface on a prism to be coated with cystamine, glutaraldehyde, and extravidin in sequence. Then, biotin-extravidin interaction was used to bind the biotinylated aptamers to the surface of the SPR chip, permitting CRP to be bound selectively to the aptamers. Meanwhile, the QDs (enhancers) surrounded by a streptavidin shell were prepared and bound to biotinylated aptamers, forming the QDs conjugated with secondary aptamers (the so-called nano-enhancers). Injection of these nano-enhancers consisting of QDs conjugated with the secondary aptamers that were CRP-specific would bind to the CRP selectively, thus enhancing both the SPR signal sensitivity and the selectivity. The SPR signal (the difference in reflectance, i.e., ∆R measured by the SPR system) changes by introducing such nano-enhancers at the various CRP concentrations (ranging from 5 fg/mL to 500 ng/mL). Injection of the nano-enhancers caused the SPR signal (∆R) to increase by a larger degree at a higher concentration with respect to the control signal. The approximately linear relationship between the concentration and the SPR signal was obtained in the range of 5 fg/mL–5 pg/mL, with the LOD of 5 fg/mL.

**Figure 5:**
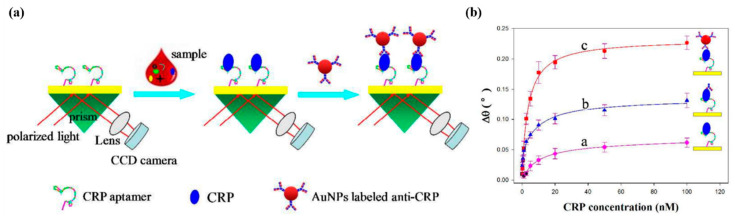
(**a**) Schematic illustration of the Au nanoparticle (AuNP)-enhanced SPR biosensor with an aptamer-antibody sandwich assay. (**b**) The relationship between Δθ and CRP concentrations by using different sensing strategies. Direct measurement a, aptamer-antibody sandwich measurement b, AuNPs enhanced aptamer-antibody sandwich measurement c. Republished with permission of Royal Society of Chemistry, from [47], 2016 copyright; permission conveyed through Copyright Clearance Center, Inc.

A hybrid sandwich assay using an aptamer and an antibody was performed for detecting CRP, with its schematic illustrated in Figure 5a [47]. First, the aptamer was immobilized onto Au surface of a SPR chip via the –SH functional group. CRP was then injected to bind to the aptamer on the Au surface. The SPR signal which was the angle shift of the darkest spot on a CCD image sensor, could be further shifted by injecting antibodies as detection elements that would bind to CRP, eventually forming a hybrid sandwich assay. In addition, replacing the detection antibody by an Au nanoparticle (AuNP)-conjugated antibody as another kind of detection element further enhances the SPR signal. This was illustrated in Figure 5b where the SPR signals were compared among three types of assays, the aptamer-CRP (direct assay), the aptamer-CRP-dAb (hybrid sandwich assay), the aptamer-CRP-AuNP conjugated dAb (another kind of hybrid sandwich assay). The CRP detection range was 1.15 ng/mL–11.5 µg/mL with the LOD of 1.15 ng/mL. We summarize the features of the SPR based CRP sensors in the Table 1.

## 3. Localized Surface Plasmon Resonance for Detecting CRP

Localized surface plasmon resonance (LSPR) occurs as a results of coherent interaction between incident light and surface electrons of metal nanoparticles at visible and near infrared wavelengths. For LSPR, their diameters need to be smaller than the incident wavelength such that the electric field of the light across the nanoparticle is nearly constant, giving rise to electron density oscillation at the incident light frequency [87]. This generates the subsequent attraction between background ions and surface electrons, and the repulsion between electrons of enhanced density. These two effects would lead to restoring force. This determines the inherent resonance frequency of the oscillation, such as in a driven harmonic oscillator model. It is noted that the nanoparticle geometry that look like a zero dimensional dot can provide the momenta required for exciting the plasmonic resonances.

The oscillating plasmons would re-radiate optical energy of light in the form of elastic scattering with its cross section much larger than the physical cross section of a nanoparticle [88]. In addition, Ohmic loss occurs due to electron-electron scattering into heat. These radiative and non-radiative damping effects lead to optical extinction of light that propagates through an ensemble of nanoparticles. The peak of the extinction spectrum with its broadening properties would be determined by the type/size/shape of metal nanoparticles, and the molecules adjacent to them. Molecules in close proximity to nanoparticles can be polarized into additional electric dipole moments, weakening the restoring force (dielectric screening effects) [87]. This accounts for red shift of the extinction spectrum peak, enabling the metal nanoparticles to lend themselves to use for sensing bio-interaction and bonding. This working principle of sensing is strongly supported by the plasmonic enhancement of electric field strength around nanoparticles, which would be much higher than that of the incident field despite its locality in space within 10 nm from the particle surface [89,90].

**Figure 6:**
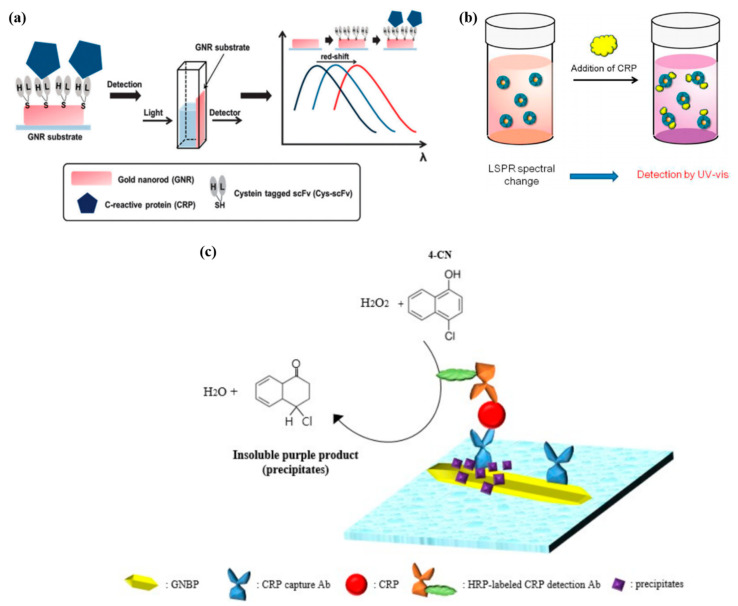
(**a**) Schematic illustration of label-free detection of CRP using gold nanorods as localized surface plasmon resonance (LSPR) sensor. Republished with permission of Royal Society of Chemistry, from [49], 2013 of copyright; permission conveyed through Copyright Clearance Center, Inc. (**b**) CRP nanosensing based on LSPR property of PMPC-g-AuNPs by ultraviolet–visible (UV–Vis) spectrophotometer. Reprinted with permission from [50] Copyright 2014 American Chemical Society. (**c**) Illustration of the LSPR immunosensor for CRP detection using 4-CN precipitation on GNBP substrate. Reproduced with permission from [51]; published by SPIE, 2017.

The CRP detection was demonstrated in a direct immunoassay by exploiting the peak shifts of extinction spectra of light transmitting through an ensemble of AuNPs). This detection scheme used the single chain variable fragment (scFv) tagged with cysteine as the CRP receptors immobilized on a gold nanorod (GNR) substrate as shown in Figure 6a [49]. The reduced size of scFv compared to that of a full-length antibody would permit CRP to more strongly interact with the LSPR evanescent field that had limited penetration depth, accounting for the detection limit as low as 1 ng/mL.

Use of a polymer based artificial receptor for CRP capture was demonstrated in the LSPR based detection [50]. The poly(2-methacryloyloxyethyl phosphorylcholine)-grafted AuNPs (PMPC-g-AuNPs) were synthesized as the substrate that underwent LSPR changes as a result of CRP attachment as illustrated in Figure 6b. The extinction spectra of light through the PMPC-g-AuNPs were analyzed using the peak-area ratio-metric sensing [91,92,93]. In this analysis, the measured spectral absorbance was numerically integrated over two different ranges of wavelengths, e.g., 490–540 nm (dispersed state), and 550–700 nm (aggregated state), into the two values A and D, respectively. The ratio A/D represents the characteristic of the extinction spectrum. Thus, injection of CRP could change the extinction spectra, thereby modifying the ratio A/D. In contrast, injection of human serum albumin (HSA) as the protein references that contained proteins that would bind to the receptor non-specifically, caused little change in the spectra. Eventually the PMPC-g-AuNPs based sensing system enabled CRP to be detected specifically with the detection limit of 50 ng/mL (detection range of 10 ng/mL–3 µg/mL).

The sandwich immunoassay with horseradish peroxide (HRP) found in the ELISA could be utilized to detect CRP in a LSPR-based sensing system as shown in Figure 6c, gold nano-bipyramid (GNBP) was used as a substrate on which CRP capture antibody was immobilized by physisorption. CRP was injected to bind the capture antibody [51]. The HRP-labeled detection antibody was then injected to bind CRP, followed by reduction of 4-chloro-1-naphthol (4-CN) by the HRP catalyst with hydrogen peroxide. The 4-CN precipitated near GNBP, thus changing the extinction spectrum of light through them. The increase in CRP concentration induced the redshift, producing the detection range of 100 pg/mL–100 ng/mL with the detection limit of 87 pg/mL. We summarize the characteristics of the LSPR-based CRP detection sensors reviewed in Table 2.

## 4. Discussion

Plasmonic optical biosensors are reviewed with a focus on those developed for highly sensitive and specific detection of CRPs, the important biomarkers not only for general inflammatory responses, but also for cardiovascular diseases and neurological disorders. This review categorizes the plasmonic biosensors into two kinds, i.e., those on SPR occurring between a flat metal film and dielectric analyte media, and those using LSPR occurring on metal nanostructures with their size much smaller than incident light wavelength.

CRP immunoassays that exploited the antibodies or aptamers as recognition receptors were applied on the sensing surface of the plasmonic devices. We could divide types of the CRP immunoassay used in the plasmonic device into three kinds, i.e., the direct assay, the sandwich assay, and the sandwich assay modified with metal nanoparticles or catalyst for enhancing sensitivity. It is inferred that the limited range of penetration depth of plasmonic evanescent fields needs to be circumvented [57] to enhance sensitivity in the CRP plasmonic immunoassay without narrowing the range of detection for a quantitative assay, such as by exploiting the extended penetration depth of plasmonic evanescent fields [57,94,95,96,97,98,99,100].

It was revealed that the SPR-based sensors were more effective in detecting the CRPs at low concentration levels than the LSPR-based ones. This is due to the fact that the LSPR relied on the more limited range over which the plasmonic evanescent field extended to interact with molecules of the detection elements. However, it turned out that the LSPR-based device did not require the optical alignment as sophisticated as the SPR-based ones. Meanwhile, regeneration of the sensor surface may be more likely to be possible in the SPR-based ones than the LSPR-based ones due largely to the extraordinarily limited range of evanescent field penetration depth from the surface in the LSPR cases.

Further work may place an additional emphasis on how to enhance the coefficient of variation in CRP immunoassay for reliable detection. These can include the homogeneous distribution of immunoassay biomolecules on the plasmonic surface, minimization of the intervention of manual expertise required to implement such label-free plasmonic sensing, and the development of highly stable recognition receptors with uniform binding affinity. This optimization can possibly lead to use of the plasmonic immunoassay for small-sized label-free devices required where time-dependent CRP concentration needs to be monitored.

## Figures and Tables

**Table 1:** The characteristics of SPR-based CRP sensors reviewed.

Reference	Assay Type	Receptor	Detection Element	LOD	Detection Range	Detection Time[s]	Remark
[42]	direct assay	antibody	CRP	1 μg/mL	1~10 μg/mL	1200	
[29]	direct assay	antibody	CRP	-	1~26 μg/mL	2400	
[44]	direct assay	aptamer	CRP	5 ng/mL	5~100 ng/mL	1400	reusable
[45]	direct assay	parylene N film	CRP	-	1 ng/mL~1 μg/mL	5000	
[48]	direct assay	antibody	CRP	9 ng/mL	9 ng/mL~70 μg/mL	1200	Fiber used
[19]	sandwich assay	antibody	antibody	1 μg/mL	2~5 μg/mL	8000	
[46]	nano-enhancer-sandwich assay	aptamer	QD-antobody	5 fg/mL	5 fg/mL~5 pg/mL	11,000	
[47]	nano-enhancer-sandwich assay	aptamer	AuNP-antibody	1.15 ng/mL	1.15 ng/mL~11.5 μg/mL	-	

**Table 2:** The characteristics of LSPR-based CRP sensors reviewed.

Reference	Sensing Strategy	Receptor Type	Detection Media	LOD	Detection Range	Remark
[49]	direct assay	scFv	CRP	-	1 ng/mL~10 μg/mL	
[50]	direct assay	PMPC	CRP	50 ng/mL	10 ng/mL~3 μg/mL	
[51]	sandwich assay	antibody	precipitates	87 pg/mL	0~100 ng/mL	
